# ﻿Taxonomic revision of *Romaleosyrphus* Bigot (Diptera, Syrphidae), including descriptions of seven new species

**DOI:** 10.3897/zookeys.1075.55862

**Published:** 2021-12-07

**Authors:** Kevin M. Moran, Jeffrey H. Skevington

**Affiliations:** 1 Canadian National Collection of Insects, Arachnids and Nematodes, Agriculture and Agri-Food Canada, 960 Carling Avenue, Ottawa, ON K1A 0C6, Canada Canadian National Collection of Insects, Arachnids and Nematodes Ottawa Canada; 2 Carleton University, Department of Biology, 1125 Colonel By Drive, Ottawa Ontario K1S 5B6, Canada Carleton University Ottawa Canada

**Keywords:** Criorhinina, Eristalinae, flower fly, hoverfly, identification key, taxonomy

## Abstract

The genus *Romaleosyrphus* Bigot is reviewed, including the description of seven new species (*R.argosi* Moran, **sp. nov.**, *R.bigoti* Moran, **sp. nov.**, *R.drysus* Moran, **sp. nov.**, *R.nephelaeus* Moran & Thompson, **sp. nov.**, *R.soletluna* Moran & Thompson, **sp. nov.**, *R.vockerothi* Moran & Thompson, **sp. nov.** and *R.woodi* Moran, **sp. nov.**). *Romaleosyrphusarctophiloides* (Giglio-Tos), **comb. nov.** is transferred to *Romaleosyrphus*. *Romaleosyrphus***stat. rev.** is redefined to represent the monophyletic unit of species within Criorhinina which possess holoptic males, a proximal ventral half of vein C with setae, a broad intersection of vein R_1_ with vein C, the distal part of R_4+5_ beyond M_1_ longer than cross-vein h and appressed pile on the abdomen. Descriptions, habitus and genitalia photographs, distributions, and an illustrated key for all nine *Romaleosyrphus* are presented. DNA barcode data are provided for eight of the species with a cytochrome *c* oxidase subunit I gene tree presented and discussed.

## ﻿Introduction

*Romaleosyrphus* Bigot, 1882 are large flies of the family Syrphidae (Eristalinae, Milesiini, Criorhinina) and are Batesian mimics of *Bombus* Latreille, 1802. Williston (1892) combined the genus with *Crioprora* Osten Sacken, 1878, where it remained until Thompson (1976) combined it with *Criorhina* Meigen, 1822. *Romaleosyrphus* is Neotropical in distribution, with one described species, *R.villosus* Bigot 1882, and appears to be restricted to high elevation cloud forests. Members of this genus possess the classic anteroventrally produced face predominant throughout the subtribe Criorhinina. Little is known of their natural history, with larvae never illustrated or described, but like their relatives, immatures likely live on decaying roots, in rot holes, sap-runs, or decaying wood in general (Speight 2020).

Moran et al. (2021) resurrected *Romaleosyrphus*, as the single Neotropical species sampled was recovered sister to the genus *Matsumyia* Shiraki, 1930. Neotropical species concepts of *Criorhina* s. l. have never been reviewed. Considering this revived generic status, a detailed examination is necessary to explore species membership in the genus and to confirm that separation of *Matsumyia* from the older concept of *Romaleosyrphus* Bigot, 1882 is warranted.

In the present study we provide evidence to justify the split between *Romaleosyrphus* and *Matsumyia*, transfer *Criorhinaarctophiloides* (Giglio-Tos, 1892) to *Romaleosyrphus*, describe seven new species of *Romaleosyrphus*, provide habitus and genitalia photographs and distributions for all the species, and provide the first identification key to the group.

## ﻿Materials and methods

### ﻿Examined collections

A list of material examined is provided in Suppl. material 1. All specimens are labelled with a unique reference number, either with their unique collection number or in the format KM­MXXXX. Label data from the studied individuals were transcribed by hand into the online CNC database and can be accessed at https://cnc.agr.gc.ca/. Specimens were borrowed from the following institutions:

**AMNH**American Museum of Natural History, New York, USA;

**CNC**Canadian National Collection of Insects, Arachnids, and Nematodes, Ottawa, Ontario, Canada;

**ECO-TAP-E**Colección Entomológica de la Unidad San Cristóbal de las Casas de El Colegio de la Frontera Sur, México (Philippe Sagot and Rémy Vandame);

**EMEC**Essig Museum of Entomology, University of California, Berkeley, California, USA;

**INHS**Illinois Natural History Survey, Champaign, Illinois, USA;

**MRSN**Museo Regionale di Scienze Naturali, Torino, Italy;

**MZH**Finnish Museum of Natural History, Helsinki, Finland;

**MZLU**Lund Museum of Zoology, Lund, Sweden;

**NHMUK**Natural History Museum UK, London, United Kingdom;

**SEMC**Snow Entomological Museum Collection, University of Kansas, Lawrence, Kansas, USA;

**UCRC**Entomology Research Museum, Department of Entomology, University of California, Riverside, California, USA;

**USNM**National Museum of Natural History, Washington D.C., USA;

**WIRC**University of Wisconsin Insect Research Center, Department of Entomology, University of Wisconsin, Madison, Wisconsin, USA.

### ﻿Specimen photography, measurements, and figures

Morphological terminology follows Cumming and Wood (2017). Morphological features of some species were examined using an Olympus SZ60 and a Zeiss SteREO DiscoveryV12 stereo microscope. Whole habitus photographs of pinned specimens were taken using the base and StackShot parts of Visionary Digital Passport II system, an Olympus OM-D EM-5 Micro 4/3 camera with a 60mm f2.8 macro lens under illumination from a Falcon FLDM-i200 LED dome-light or using a Leica M205-C stereomicroscope equipped with a Leica DFC 450 module and using 0.6× (habitus) and 1.6× (genitalia) lenses. Final images were assembled using Zerene Stacker (http://zerenesystems.com/cms/stacker).

Photographs and descriptions are not restricted to primary types and represent our species concepts as a whole.

Male genitalia were detached after relaxation of specimens in a moisture chamber and then macerated in heated lactic acid overnight before examination and photography. Afterwards the lactic acid was deactivated, the genitalia stored in plastic micro vials containing glycerin, and attached to the pin of the dissected specimen.

Specimen measurements were taken using the Leica measurement module in Leica Application Suite (https://www.leica-microsystems.com/products/microscope-software/p/leica-application-suite/) and are based upon the smallest and largest specimen of each species. Body measurements represent the distance between the anterior end of the frons and the posterior end of tergite IV. Wing measurements represent the distance between the tegula and the apex of the wing. Maps include points from all specimens examined and were produced using SimpleMappr (https://www.simplemappr.net/).

In the description of primary type labels, the contents of each label are enclosed within double quotation marks (“ ”), italics denote handwriting, and the individual lines of data are separated by a double forward slash (//). At the end of each record, between square brackets ([]) and separated by a comma, the number of specimens and sex, the unique identifier or number, and the holding institution are given.

### ﻿DNA Sequencing

The right mid leg was removed from selected specimens. Legs were processed in house at the Canadian National Collection of Insects (CNC) by Scott Kelso using a modified version of the (Hajibabaei et al. 2005) protocol with custom primers (see Table 1).

**Table 1.** Cytochrome *c* oxidase I mitochondrial gene primers used in this study.

**Primer name**	**Primer design**	**Primer sequence**
Heb-F	Folmer et al. 1994	GGT CAA CAA ATC ATA AAG ATA TTG G
COI-Fx-A-R	Kelso (in prep.)	CGD GGR AAD GCY ATR TCD GG
COI-Fx-B-F	Kelso (in prep.)	GGD KCH CCN GAY ATR GC
COI-Fx-B-R	Kelso (in prep.)	GWA ATR AAR TTW ACD GCH CC
COI-Fx-C-F	Kelso (in prep.)	GGD ATW TCH TCH ATY YTA GG
COI-780R	Gibson et al. 2011	CCA AAA AAT CAR AAT ARR TGY TG

The primers, COI-Fx-A-R, B-F, B-R, and C-F are designed to sequence the standard animal DNA barcode region in three portions, labeled A, B, and C after the primers, increasing the chance of successfully sequencing heavily fragmented DNA. This enabled DNA barcoding of species for which only older material, typically considered unsuitable for DNA barcoding, was available.

Raw sequence reads were evaluated using Sequencer v5.4.6 (http://www.genecodes.com/) and aligned together with downloaded BOLD data using MAFFT v7 (Katoh and Standley 2013).

All sequence data obtained are stored online on the BOLD database (www.boldsystems.org). It is publicly accessible in the *Romaleosyrphus* (ROMALEO) dataset available at http://www.boldsystems.org/index.php/Public_SearchTerms?query=DS-ROMALEO.

### ﻿Molecular data analysis

Neighbor-joining analysis using uncorrected p-distance was used to explore morphological species concepts for ingroup taxa utilizing PAUP v4.0a168 (Swofford 2001). *Blerafallax* (Linnaeus, 1758), *Milesiavirginiensis* (Drury, 1773), *Temnostomaalternans* Loew, 1864, and *Xylotaflavifrons* Walker, 1849, which also belong to the tribe Milesiini, were used as outgroups of Criorhinina. For outgroups inside Criorhinina, we included any described species for which we possessed a DNA barcode. Pairwise distances were calculated using BOLD (see Table 2).

**Table 2.** Average intraspecific (diagonal) and interspecific (below diagonal) pairwise (p) distances (%) based on the barcode region of the mitochondrial cytochrome *c* oxidase subunit I gene of *Romaleosyrphus*.

	***R.arctophi-loides***	***R.argosi***	***R.bigoti***	***R.drysus***	***R.nephelaeus***	***R.soletluna***	***R.villosus***
***R.arctophi-loides***	–						
***R.argosi***	4.51	–					
***R.bigoti***	2.95	4.84	–				
***R.drysus***	4.26	3.05	3.99	–			
***R.nephelaeus***	3.58	3.31	3.13	2.85	0.97		
***R.soletluna***	3.68	5.36	3.04	4.52	3.58	0.93	
***R.villosus***	2.34	5.23	1.55	3.98	3.28	3.45	–
***R.vockerothi***	2.81	4.71	1.52	3.62	3.50	3.41	2.17

Taxa in the tree are labeled in the following format BOLD Process ID | Taxon Name | Institution Sample ID.

## ﻿Results

### ﻿Taxonomy and systematics

#### ﻿ Romaleosyrphus

Taxon classificationAnimaliaDipteraSyrphidae

Bigot, 1882

2CA9D058-340D-597F-8404-A24B51180914

Figures 1 , 2 , 3

Romaleosyrphus Bigot, 1882a: 159. –Bigot (1882b): cxxix; –Bigot (1883): 356. Type species: Romaleosyrphusvillosus Bigot, 1882 by original designation.
Rhomaleosyrphus Rye, 1884: 10. –Kertész (1910): 291. Unjustified emendation of Romaleosyrphus.
Crioprora Williston, 1891: 73. –Aldrich (1905): 401. –Coquillett (1910): 528.
Criorhina Thompson, 1976: 118.

##### Differential diagnosis.

*Romaleosyrphus* is separated from *Criorhina* and *Sphecomyia* by the combination of the following characters. Male eye contiguous for ca. 1/2 length of ocellar triangle. Oval shaped postpedical. Broad intersection of vein R_1_ with vein C. Proximal ventral half of vein C with setae. Abdominal pile appressed. Male genitalia with phallapodeme keeled and laterally sclerotized, not banana-shaped. It is further distinguished from *Matsumyia* by a distal part of vein R_4+5_ beyond vein M_1_ longer than cross-vein h.

##### Redescription.

**MALE.** Body length: 13.0–17.1 mm. Wing length: 8.0–12.1 mm.

***Head.*** Face black, produced downwards and completely pruinose, concave beneath antenna, tuberculate; gena broad, as broad or broader than long, bare, shiny, pilose posteriorly; anterior tentorial pit short, extending along ventral third of eye, pilose; frontal prominence distinct; frons broad and pruinose; vertex triangular, longer than broad and always pilose; ocellar triangle small; eye bare, contiguous for ca. 1/2 length of ocellar triangle; head oval in shape; length of antenna segments in a 3:3:2 ratio; postpedical oval, with bare arista dorsally placed.

**Figure 1.**
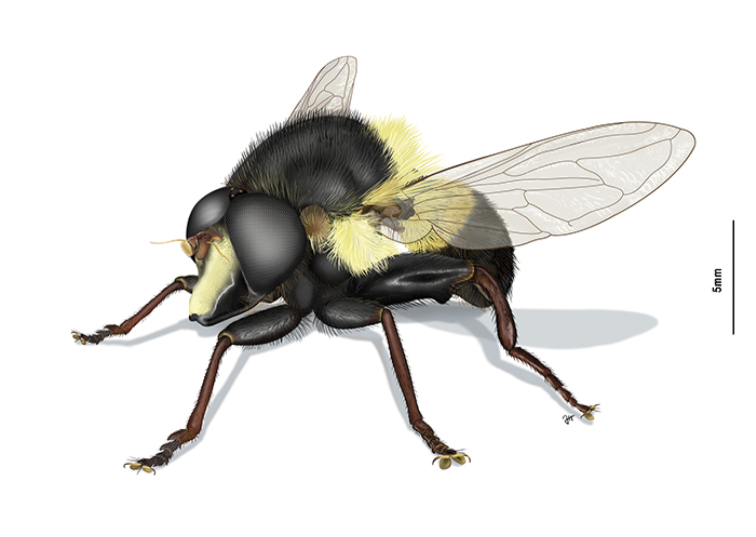
*Romaleosyrphusbigoti* sp. nov.

***Thorax*.** Ca. as long as broad, long pilose; postpronotum pilose; proepimeron pilose; anterior anepisternum bare, posterior anepisternum pilose; scutellum without apical sulcus and with ventral pile fringe; katepisternum bare anteriorly, discontinuously pilose posteriorly with broadly separated patches; anepimeron with anterior portion pilose, and dorsomedial and posterior portion bare; katepimeron bare; metathoracic pleuron bare; without hypopleural pile at the base of the posterior thoracic spiracle; meron bare; metathoracic spiracle ca. same size as postpedical; metasternum pilose; postmetacoxal bridge incomplete; plumula simple, elongate, short, not reaching calypteral margin; calypter brown.

**Figure 2.**
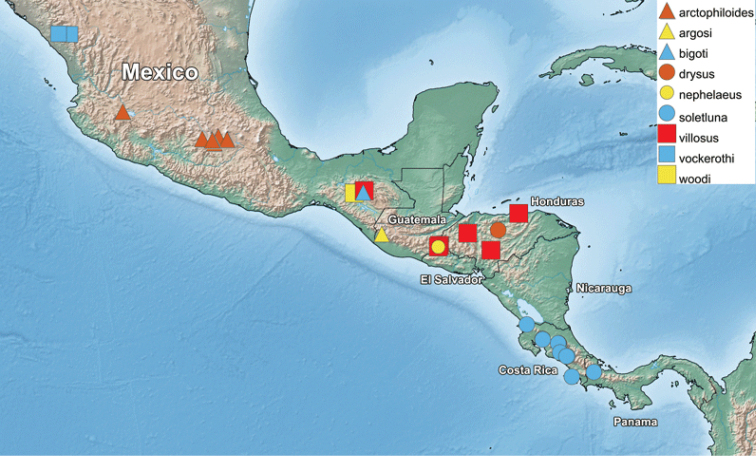
*Romaleosyrphus* distribution.

***Legs*.** Coxae pilose anteriorly, bare posteriorly; hind trochanter sometimes tuberculate in male; metafemur swollen, curved, with large apicoventral ridge and without basiventral setose patch; metatibia transverse apically, rounded basiventrally.

**Figure 3.**
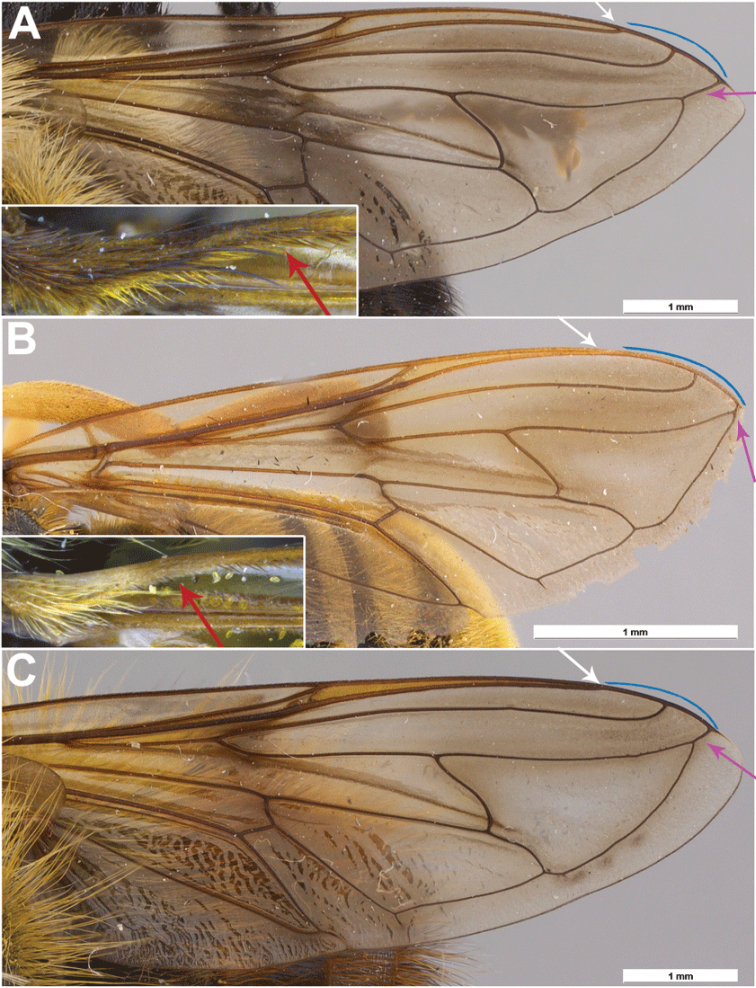
Intersection of vein R_1_ with vein C (white), distance between apices of veins R_1_ and R_2+3_ and apices of veins R_2+3_ and R_4+5_ (blue), distal vein R_4+5_ (pink) and setosity of proximal ventral half of vein C (red). **A***Romaleosyrphusbigoti* sp. nov. **B***Sphecomyiaweismani* (Moran) **C***Criorhinabubulcus* (Walker).

***Wing*.** Cell r_1_ open; stigmatic cross vein present; cross-vein r-m at outer ¼ of cell dm; broad intersection of vein R_1_ with vein C (Fig. 3); vein R_4+5_ straight; distance between apices of veins R_1_ and R_2+3_ longer than distance between apices of veins R_2+3_ and vein R_4+5_; distal part of vein R_4+5_ beyond vein M_1_ (hereafter distal vein R_4+5_) longer than cross-vein h (Fig. 3); vein M_2_ absent; vein CuP+CuA short, curved; proximal ventral half of vein C with setae.

***Abdomen*.** Oval, slightly longer than broad, with dense appressed pile.

***Male genitalia*.** Surstyli symmetric; aedeagus segmented, with phallapodeme separated from basiphallus and distiphallus; phallapodeme rounded, not banana-shaped; well-developed ctenidion present in male genitalia.

**FEMALE.** As male except for the following character states. Eyes widely separated; frons fully brown pruinose; face without pruinosity; metafemur only slightly swollen, never curved or with apicoventral ridge; metatibia never modified; always without tubercle on hind trochanter; wing always less microtrichose with species-specific characters as in species description.

##### Remarks.

Generally, species of *Romaleosyrphus* show little variation in pile color patterns, at least given the limited material we worked with. However, there are a few exceptions. *Romaleosyrphussoletluna* Moran & Thompson, sp. nov. is drastically dimorphic in pile coloration with a mostly orange morph and mostly black morph. The single northern specimen of *Romaleosyrphusarctophiloides* from the Sierra Madre Occidental has fully black pilose legs. This contrasts with the population surrounding Mexico City, from which the type was collected, which have a streak of yellow pile at the base of the fore and mid femora. Finally, pile color on the proepimeron is variable inside multiple species with observed character states being fully yellow, fully black or a mix of the two. We suspect that additional material will likely show proepimeron pile color to be variable in all species.

### ﻿Key to *Romaleosyrphus* species

**Table:** 

1	Scutellum entirely black pilose, with only a few posterolateral yellow pile at most; post-alar callus extensively black pilose; male hind tibia as in Fig. 9D; male genitalia as in Fig. 11B	***R.soletluna* Moran & Thompson, sp. nov.**
–	Scutellum partially yellow pilose; post-alar callus extensively yellow pilose	**2**
2	Scutellum entirely rufous or yellow pilose	**4**
–	Scutellum black pilose medially	**3**
3	Tergite II–III extensively rufous to yellow pilose; male hind trochanter not tuberculate (Fig. 8A); male hind tibia as in Fig. 9A; male genitalia as in Fig. 11A	***R.arctophiloides* (Giglio-Tos)**
–	Tergite II black pilose on posterolateral corners; Tergite III black pilose except yellow pilose anteromedially; male unknown but hind trochanter likely tuberculate (Fig. 8B)	***R.woodi* Moran, sp. nov.**
4	Tergite III extensively black pilose	**6**
–	Tergite III extensively rufous to yellow pilose	**5**
5	Mesonotum entirely yellow to rufous pilose; male hind tibia as in Fig. 9D; male genitalia as in Fig. 11B	***R.soletluna* Moran & Thompson, sp. nov.**
–	Mesonotum extensively black pilose medially; male hind tibia as in Fig. 9E; male genitalia as in Fig. 11D	***R.vockerothi* Moran & Thompson, sp. nov.**
6	Tergite IV extensively yellow pilose; male hind tibia as in Fig. 9C; male genitalia as in Fig. 11C	***R.nephelaeus* Moran & Thompson, sp. nov.**
–	Tergite IV entirely black pilose	**7**
7	Tergite II without black pile	**9**
–	Tergite II with conspicuous black pile	**8**
8	Tergite II extensively white pilose, extending from anterolateral corners to posteromedial edge	***R.argosi* Moran, sp. nov.**
–	Tergite II black pilose except yellow pilose in anterolateral corners and along the posterior rim	***R.drysus* Moran, sp. nov.**
9	Tergite II rufous pilose posteriorly; tergite III rufous pilose anteriorly; male hind tibia as in Fig. 9E; male genitalia as in Fig. 11F	***R.villosus* Bigot**
–	Tergite II without rufous pile; tergite III entirely black pilose; male hind tibia as in Fig. 9B; male genitalia as in Fig. 11E	***R.bigoti* Moran, sp. nov.**

#### ﻿ Romaleosyrphus arctophiloides

Taxon classificationAnimaliaDipteraSyrphidae

(Giglio-Tos, 1892) comb. nov.

F7344727-BD36-524F-8F12-694695D8B0D2

**Figure 4.**
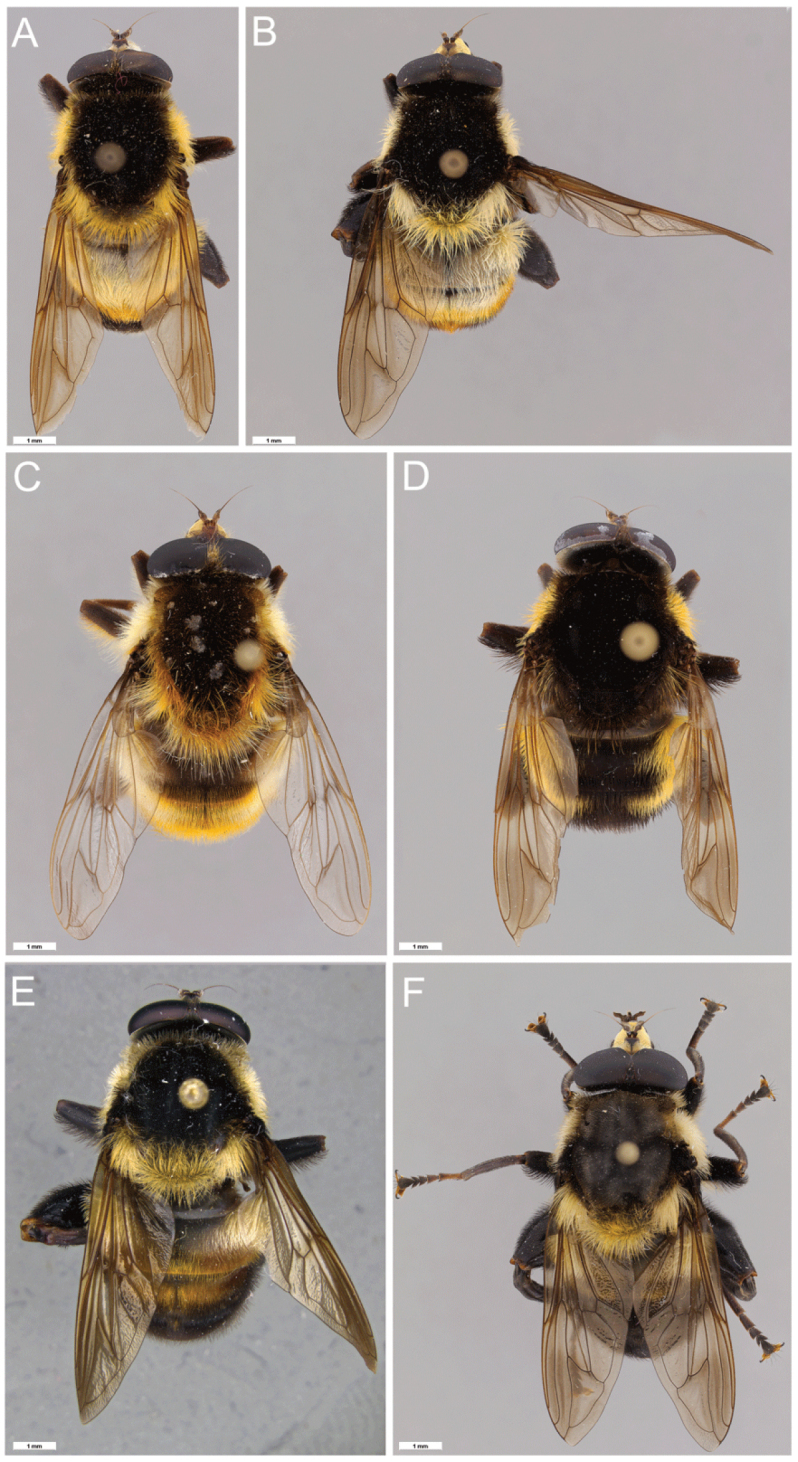
*Romaleosyrphus* dorsal habitus **A**: *Romaleosyrphusarctophiloides***B**: *Romaleosyrphusvockerothi* sp. nov. **C**: *Romaleosyrphussoletluna* sp. nov. rufous morph **D**: *Romaleosyrphussoletluna* sp. nov. black morph **E**: *Romaleosyrphusvillosus***F.***Romaleosyrphusbigoti* sp. nov.

Figures 4A , 6A , 8A , 9A , 10A , 11A

Crioprora arctophiloides Giglio-Tos, 1892: 7. –Giglio-Tos (1893): 25. –Aldrich (1905): 401. Type locality: Mexico, Angang[ueo] [MRSN]
Penthesilea arctophiloides Kertész, 1910: 286.
Criorhina tapeta Fluke, 1939: 369. –Thompson (1976): 119. Type locality: Mexico City, 10,000 ft. [AMNH]
Criorhina arctophiloides Thompson, 1976: 118.

##### Material examined.

**Mexico**. **Durango**: 14 miles Southwest of El Salto, 23.702772, -105.564053, 2438m, 30.vi.1964, W.R.M. Mason, CNC_Diptera142464 (1♂, CNC); **Mexico City, D.F.**: San Pedro Atocpan, 19.204792, -99.048853, 2600m, 16.ix.1947, C. Bolivar, CNC_Diptera142465 (1♂, CNC);1910, USNM_ENT1071372 (1♂, USNM); **Mexico**: Edo. de Mexico, km. 73rd to Popocatépetel, 19.075366, -98.65902, 3352m, 15.vii.1961, D.H. Janzen, EMEC354664 (1♀, EMEC); Nevado Toluca, 19.110036, -99.753425, 3200m, 11.vii.1951, H.E. Evans, Jeff_Skevington_Specimen52560 (1♂, CNC); 19.110035, -99.753423, 3444m, 11.vii.1951, P.D. Hurd, EMEC354662 (1♂, EMEC); West Slope, Cortez Pass, 19.08569, -98.648296, 2743m, 13.vii.1954, R.R. Dreisbach, KMM0919 (1♂, WIRC);19.08569, -98.648296;19.085692, -98.648297, 2743m;~13.vii.1954, CNC_Diptera142466;CNC_Diptera142467 (1♂, 1♀, CNC); Mexico City, 19.42250, -99.14389, 10000ft, vii.1936 (1♂ HT AMNH); **Morelos**: #17 Lagunas de Zempoala Nat. Park, 19.04828, -99.312179, 2865m, 23.viii.1969, G.W. Byers, KMM0920 (1♂, SEMC); Cuernavaca, 18.924211, -99.221567, 2133m, 29.vii.1961, R. & K. Dreisbach, J_Skevington_Specimen50177 (1♀, ANSP).

##### Differential diagnosis.

Scutellum only partly yellow pilose, black pilose anteriorly and medially. Tergite II–III extensively rufous to yellow pilose. Tergite IV dominantly black pilose, but sometimes with rufous or yellow pile medially or posteriorly. Hind trochanter not tuberculate in male.

##### Redescription.

**MALE.** Body length: 13.1–14.8 mm. Wing length: 8.6–9.4 mm.

***Head*.** Face shape as in Fig. 10A; face silver or gold pruinose; gena black pilose posteriorly; anterior tentorial pit variable pilose: yellow or black; frons broad, ca. as long as broad at antenna, 2/3 as broad at vertex as at antenna, black pilose and silver-gold pruinose; vertex triangular, longer than broad, black pilose and brown pruinose; postocular setae black; occipital setae variable: yellow or black; antenna reddish orange.

***Thorax.*** Matte black; postpronotum variable pilose: black or mixed black and yellow; scutum black pilose; scutellum yellow pilose, except black pilose anteromedially; postalar callus variable pilose: yellow, black or mixed black and yellow; proepimeron black pilose; posterior anepisternum yellow pilose; katepisternum yellow pilose posteriorly with broadly separated patches; metasternum variable pilose: black, yellow or mixed black and yellow; anepimeron with anterior portion yellow pilose; lower calypter with long black pile.

***Legs.*** Coxae black; femora black except extreme apex of femora; remainder of legs reddish; hind trochanter rounded, not tuberculate as in Fig. 8A; fore and mid-coxae black pilose; hind coxa mixed black and yellow pilose; fore femur black pilose, except occasionally with small mix of yellow pile basally; mid femur fully black pilose or with stretch of yellow pile on posterior side; hind femur black pilose; tibiae and tarsi black pilose; hind tibia as in Fig. 9A.

***Wing*.** Microtrichia absent from following areas: broad anterior margin of cell cua.

***Abdomen*.** Tergites shiny to subshiny black; tergite I with scattered, yellow pile medially, except with short black pile in lateral corners; tergite II with dense yellow pile; tergite III with dense pile which is yellow anteromedially, rufous from anterolateral corners to posteromedial margin and black in posterolateral corners; tergite IV variable, dominantly black pilose, but sometimes with rufous or yellow pile medially or posteriorly; grey pruinosity as follows: tergite I pruinose posteriorly, all of tergite II, tergite III except in posterolateral corners; sternites I–III yellow pilose and not pruinose; sternite IV variable: black or rufous pilose or some mix of the two; pile of postabdomen rufous or yellow.

***Male genitalia*.** (Fig. 11A) Cercus yellowish brown, broader at apex, covered with long yellow pile; surstylus brown, ca. 2 × as long as broad, broadened basally with apical third tapering, directed ventrally and with an acute apex, ventral margin concave, undulated; pile on dorsal surface of surstylus, increasing in length posteriorly; minute spines on ventral surface and apical 3/4^th^ of lateral inner and outer surface.

**FEMALE.** As male, except for usual sexual dimorphism; microtrichia on wing absent in following areas: broad anterior margin of cell cua, medial area of cell bm, anterior margin in cell dm, small region anteriorly in cell m_4_ near cross-vein m-cu.

##### Distribution.

Mexico.

##### Habitat.

Trans-Mexican Volcanic Belt pine-oak forests ecoregion.

##### Remarks.

*Romaleosyrphusarctophiloides* is the only known member of *Romaleosyrphus* in which the hind trochanter is not tuberculate in the male. Although males are not known for *Romaleosyrphusargosi* sp. nov., *R.drysus* sp. nov. and *R.woodi* sp. nov., males of their closest relative in the COI gene tree, *R.nephelaeus* sp. nov., possess a tuberculate hind trochanter. It is therefore expected that males of these three species also have a tuberculate hind trochanter.

We suspect that a single specimen “CNC_Diptera142464” collected in the Sierra Madre Occidental may represent a distinct species from specimens collected in the Trans-Mexican Volcanic Belt pine-oak forests. Although no genital or discrete morphological differences could be found, the legs of this specimens are fully black pilose while those of all the others have a streak of yellow pile at the base of the fore and mid femora. Unfortunately, while a barcode was obtained for this specimen, no barcode sequences were obtained from specimens from specimens collected in the Trans-Mexican Volcanic Belt pine-oak forests.

#### ﻿ Romaleosyrphus argosi

Taxon classificationAnimaliaDipteraSyrphidae

Moran sp. nov.

CCCBB498-79EC-58AA-AC15-42865BCB0C1D

http://zoobank.org/0DC38597-3C3D-4846-AB0D-32DB952E3E43

Figures 5D , 7D

##### Type locality.

**Guatemala**: **San Marcos**: Bojonal Rd., 1.3 km, 14.9333, -91.8667, 1600m.

##### Types.

***Holotype*** female, pinned. Original label: “Guatemala: San Marcos // km 1.3, Bojonal Road // 14° 56’N 91° 52’W 1600m // 13-14. vii. 2001 DCH, DY” “Univ. Calif. Riverside // Ent. Res. Museum // UCRC ENT 66852” (UCRC).

**Differential diagnosis.** Scutellum white pilose. Tergite II extensively white pilose, except with black pile in posterolateral corners. Tergite III black pilose, except with mixed white pile anteromedially. Tergite IV black pilose.

##### Description.

**FEMALE.** Body length: 12.5 mm. Wing length: 8.1 mm.

***Head*.** Face non-pruinose; gena black pilose anteriorly; anterior tentorial pit black pilose; frons, black pilose and brown pruinose; vertex black pilose and brown pruinose; postocular setae black; occipital setae black; antenna reddish orange.

***Thorax.*** Matte black; postpronotum white pilose; scutum white pilose along margins and black pilose medially; scutellum white pilose; postalar callus white pilose; proepimeron black pilose; posterior anepisternum white pilose; katepisternum white pilose posteriorly with broadly separated patches; metasternum mixed black and white pilose; anepimeron with anterior portion white pilose; lower calypter with long black pile.

***Legs.*** Coxae black; femora black except extreme apex of femora; remainder of legs reddish; fore and mid-coxae black pilose; hind coxa mixed black and white pilose; fore femur black pilose, except small mix of white pile basally; mid femur black pilose, but with stretch of white pile on posterior side; hind femur black pilose; tibiae and tarsi black pilose.

***Wing*.** Microtrichia absent in following areas: cell c along margin of vein Sc running from 2/5 and ending at 4/5 of length of the cell, anterior 1/5 of cell r_1_, cell br except along spurious vein the part right below the start of cell r_2+3_, all of cell cua except extreme posterior, cell bm, cell cup along the margin of vein CuP in the anterior third of cell, cell m_4_ from cross-vein m-cu to end of vein M_4_ and in following regions of cell dm: anterior ¼, except extreme anterodorsal corner, ventral 1/3, and broad margin adjacent to vein M_2_.

**Figure 5.**
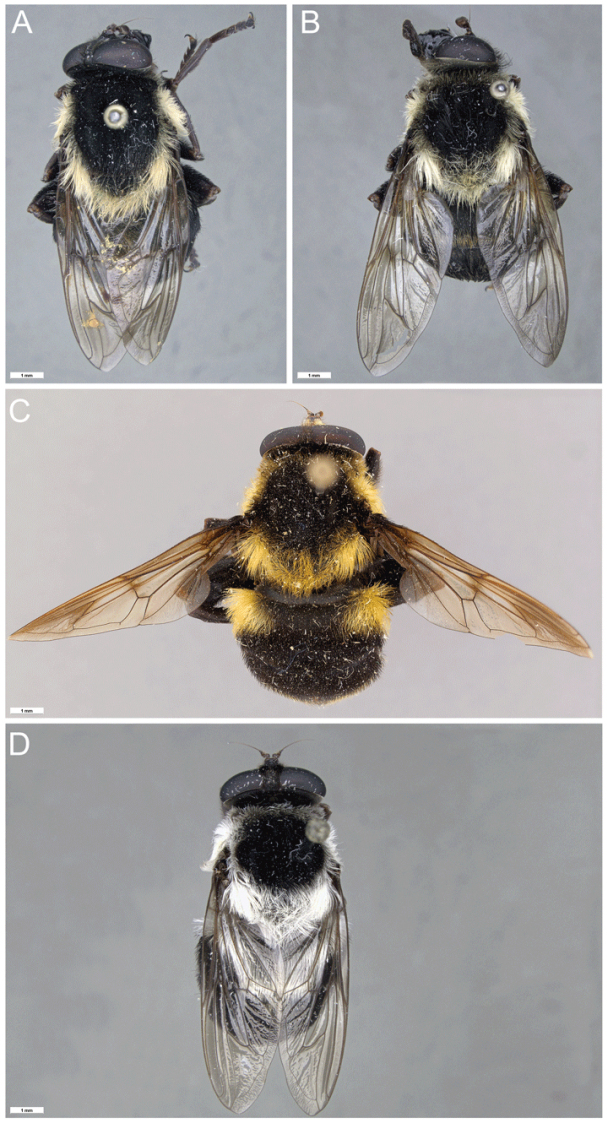
*Romaleosyrphus* dorsal habitus (cont.) **A***Romaleosyrphuswoodi* sp. nov. **B***Romaleosyrphusdrysus* sp. nov. **C***Romaleosyrphusnephelaeus* sp. nov. **D***Romaleosyrphusargosi* sp. nov.

***Abdomen*.** Tergites shiny to subshiny black; tergite I with scattered, white pile medially, except with short black pile in lateral corners; tergite II with dense white pile which runs diagonally from anterolateral corner until it reaches the posterior margin at a point which is ca. at 1/3 width of the tergite, remainder of tergite is black pilose; tergite III with black pile except mixed white pile anteromedially; tergite IV with black pile; tergites not distinctly pruinose; sternites I–III white pilose and not pruinose; sternite IV black pilose; pile of postabdomen black.

**MALE.** Unknown.

##### Distribution.

Guatemala.

##### Habitat.

Central American montane forests ecoregion.

##### Etymology.

Named *argosi*, from the Greek *argos* (white), to highlight the coloration of this species. It is a noun in apposition.

#### ﻿ Romaleosyrphus bigoti

Taxon classificationAnimaliaDipteraSyrphidae

Moran sp. nov.

CCAF31CC-3EF3-5144-B245-472D3928426C

http://zoobank.org/F9ABF7C4-900A-42A1-9E33-B5D397AC1B39

**Figure 6.**
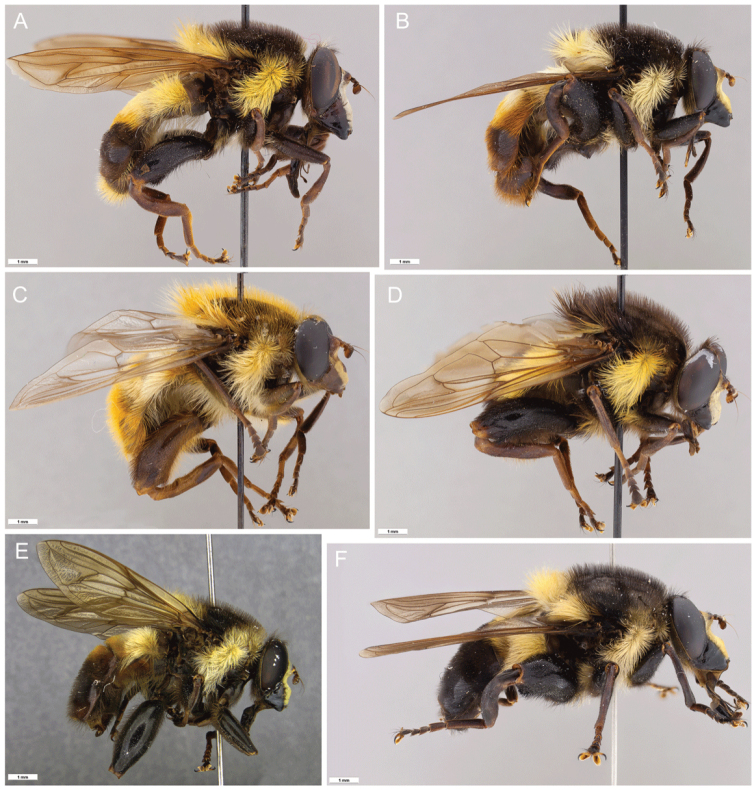
*Romaleosyrphus* lateral habitus **A***Romaleosyrphusarctophiloides***B***Romaleosyrphusvockerothi* sp. nov. **C***Romaleosyrphussoletluna* sp. nov. rufous morph **D***Romaleosyrphussoletluna* sp. nov. black morph **E***Romaleosyrphusvillosus***F***Romaleosyrphusbigoti* sp. nov.

Figures 3A , 4F , 6F , 9B , 10B , 11E

##### Type locality.

**Mexico**: **Chiapas**: San Cristóbal de las Casas, Huitepec, 16.7603, -92.6814, 2560m.

##### Types.

***Holotype*** male, pinned. Original label: “Mexico-Chiapas // San-Cristobal-de-las-Casas // Huitepec Alt: 2560m. // N16°44’35”/W92°41’17” // 9-02-2009 // SAGOT P. n°7” “Diptera-Brachycera // Syrphidae // Criorhina sp. 1 // Male // Coll. SAGOT P. **n°1016**” “J. Skevington // Specimen # // 52561” (ECO-TAP-E).

**Differential diagnosis.** Scutellum yellow pilose. Tergite II completely yellow pilose. Tergite III black pilose. Tergite IV black pilose. Male hind tibia as in Fig. 9B. Male genitalia as in Fig. 11E.

##### Description.

**MALE.** Body length: 15.2 mm. Wing length: 10.5 mm.

***Head*.** Face shape as in Fig. 10B; face gold pruinose; gena black pilose posteriorly; anterior tentorial pit variable pilose: yellow or black; frons broad, ca. as long as broad at antenna, 2/3 as broad at vertex as at antenna, black pilose and silver-gold pruinose; vertex triangular, longer than broad, black pilose and brown pruinose; postocular setae black; occipital setae variable: yellow or black; antenna reddish orange.

***Thorax.*** Matte black; postpronotum mixed black and yellow pilose; scutum black pilose; scutellum yellow pilose; postalar callus yellow pilose; proepimeron yellow pilose; posterior anepisternum yellow pilose; katepisternum yellow pilose posteriorly with broadly separated patches; metasternum mixed black and yellow pilose; anepimeron with anterior portion yellow pilose; lower calypter with long black pile.

***Legs.*** Coxae black; femora black except extreme apex of femora; remainder of legs reddish; hind trochanter tuberculate as in Fig. 8B; fore and mid-coxae black pilose; hind coxa mixed black and yellow pilose; fore femur black pilose, except small mix of yellow pile basally; mid femur black pilose, but with stretch of yellow pile on posterior side; hind femur black pilose; tibiae and tarsi black pilose; hind tibia as in Fig. 9B.

***Wing*.** Microtrichia absent from following areas: broad anterior margin of cell cua.

***Abdomen*.** Tergites shiny to subshiny black; tergite I with scattered, yellow pile; tergite II with dense yellow pile; tergite III with black pile; tergite IV with black pile; grey pruinosity as follows: tergite I pruinose posteriorly, all of tergite II pruinose; sternites I–III yellow pilose and not pruinose; sternite IV black pilose; pile of postabdomen black.

**Figure 7.**
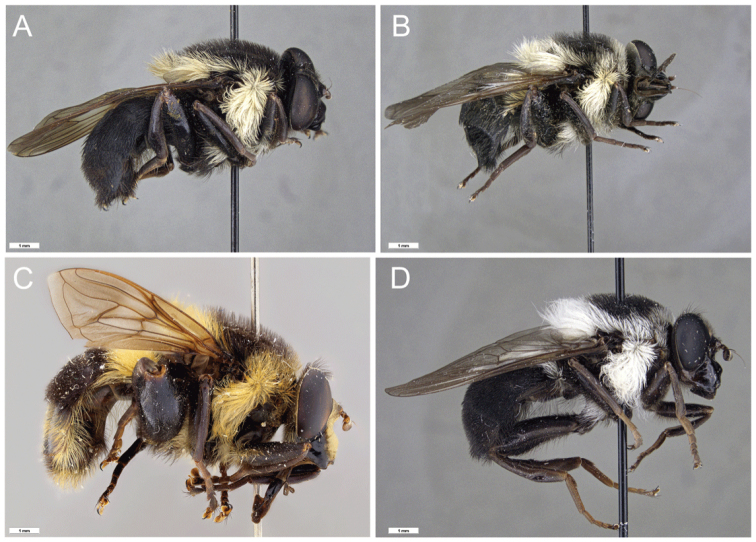
*Romaleosyrphus* lateral habitus (cont.) **A***Romaleosyrphuswoodi* sp. nov. **B***Romaleosyrphusdrysus* sp. nov. **C***Romaleosyrphusnephelaeus* sp. nov. **D***Romaleosyrphusargosi* sp. nov.

***Male genitalia*.** (Fig. 11E) Cercus yellowish brown, broader at apex, covered with long yellow pile; surstylus brown, ca. 4 × as long as broad, broadened basally with apical ha1/2lf tapering, directed ventrally and with a rounded apex, ventral margin concave, undulated; pile on dorsal surface of surstylus, increasing in length posteriorly; minute spines on ventral surface and apical 3/4 of lateral inner and outer surfaces.

**FEMALE.** Unknown.

##### Distribution.

Mexico.

##### Habitat.

Central American pine-oak forests ecoregion.

##### Etymology.

Named after Bigot who erected this genus in 1882.

#### ﻿ Romaleosyrphus drysus

Taxon classificationAnimaliaDipteraSyrphidae

Moran sp. nov.

783C897C-071A-5567-9AB0-A88C8AF07055

http://zoobank.org/10B87EF5-2E8A-457F-9F58-AB34F235E66E

Figures 5B , 7B

##### Type locality.

**Honduras**: La Muralla National Park, vicinity of visitor center, 15.1058, -86.7528, 1460m.

##### Types.

***Holotype*** female, pinned. Original label: “HONDURAS: Olancho // La Muralla National Park // vicinity of Visitor Center // 1460 m; 9-13 May 1999 // D.C. Hawks & J. Torres” “Univ. Calif., Riverside // Ent. Res. Museum // UCRC ENT 00035151” (UCRC).

**Differential diagnosis.** Scutellum entirely yellow pilose. Tergite II black pilose except yellow pilose in anterolateral corners and along the posterior rim. Tergite III extensively black pilose.

**Figure 8.**
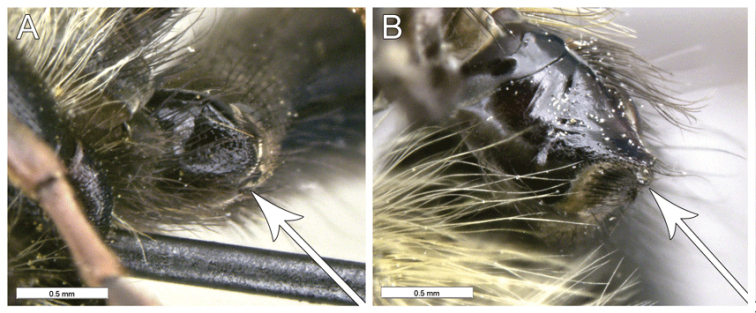
*Romaleosyrphus* 3^rd^ trochanter tubercle **A***Romaleosyrphusarctophiloides***B***Romaleosyrphusvillosus*.

**Description. FEMALE.** Body length: 13.4 mm. Wing length: 8.9 mm.

***Head*.** Face non-pruinose; gena black pilose anteriorly; anterior tentorial pit black pilose; frons, black pilose and brown pruinose; vertex black pilose and brown pruinose; postocular setae black; occipital setae black; antenna reddish orange.

***Thorax.*** Matte black; postpronotum yellow pilose; scutum yellow pilose along margins and black pilose medially; scutellum yellow pilose; postalar callus yellow pilose; proepimeron yellow pilose; posterior anepisternum yellow pilose; katepisternum yellow pilose posteriorly with broadly separated patches; metasternum mixed black and yellow pilose; anepimeron with anterior portion yellow pilose; lower calypter with long black pile

***Legs.*** Coxae black; femora black except extreme apex of femora; remainder of legs reddish; fore and mid-coxae black pilose; hind coxa mixed black and yellow pilose; fore femur black pilose, except small mix of yellow pile basally; mid femur black pilose, but with stretch of yellow pile on posterior side; hind femur black pilose; tibiae and tarsi black pilose.

***Wing*.** Microtrichia absent in following areas: cell c along margin of vein Sc running from 2/5 and ending at 4/5 of length of the cell, cell br except along margins of cell and along spurious vein and the part right below the start of vein r_2+3_, all of cell cua except extreme posterior, ventral half of cell bm, cell m_4_ from cross-vein m-cu to end of vein M_4_ and cell dm in ventral 1/3 of cell and along broad margin following vein M_2._

***Abdomen*.** Tergites shiny to subshiny black; tergite I with scattered, yellow pile medially, except with short black pile in lateral corners; tergite II with dense yellow pile on anterior 2/3 and black pile on anterior third; tergite III with black pile; tergite IV with black pile; tergites not distinctly pruinose; sternites I–III yellow pilose and not pruinose; sternite IV black pilose; pile of postabdomen black.

**Figure 9.**
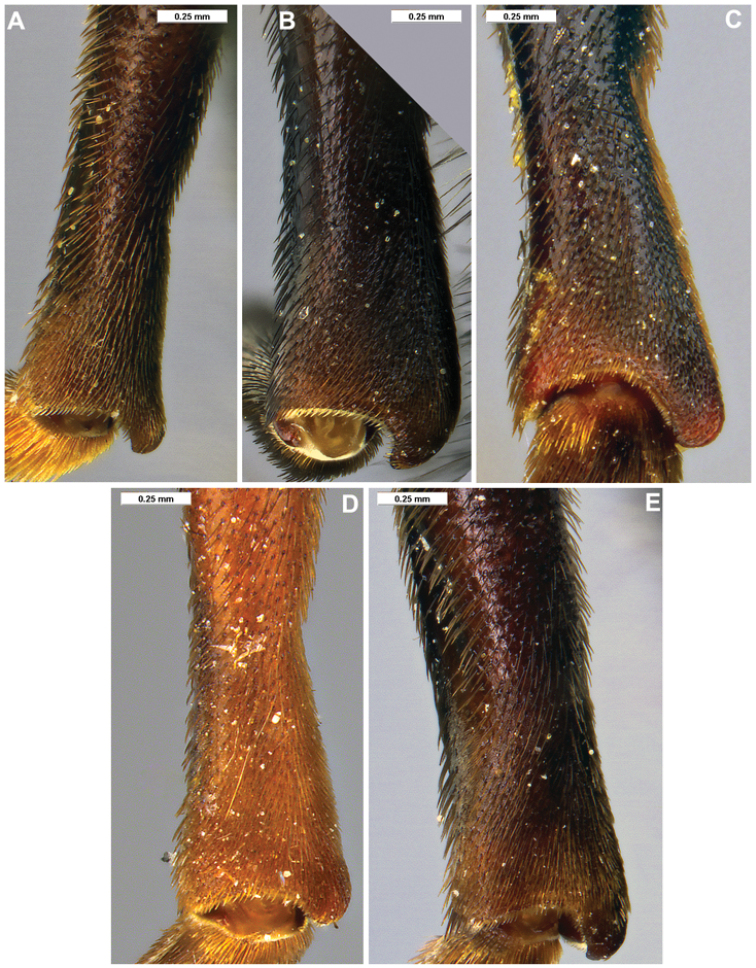
*Romaleosyrphus* male hind tibia **A***Romaleosyrphusarctophiloides***B***Romaleosyrphusbigoti* sp. nov. **C***Romaleosyrphusnephelaeus* sp. nov. **D***Romaleosyrphussoletluna* sp. nov. **E***Romaleosyrphusvillosus*.

**MALE.** Unknown.

##### Distribution.

Honduras.

##### Habitat.

Central American montane forests ecoregion.

##### Etymology.

Named *drysus*, derived from the Greek *drys* for oak, in reference to the high elevation oak forests this species lives in. It is a noun in apposition.

**Figure 10.**
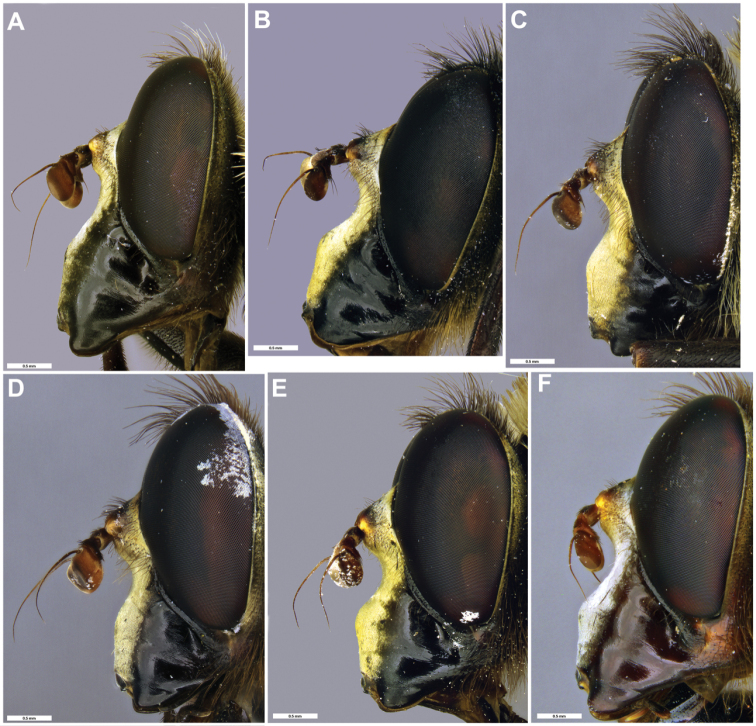
*Romaleosyrphus* male face **A***Romaleosyrphusarctophiloides***B***Romaleosyrphusbigoti* sp. nov. **C***Romaleosyrphusnephelaeus* sp. nov. **D***Romaleosyrphussoletluna* sp. nov. **E***Romaleosyrphusvillosus***F***Romaleosyrphusvockerothi* sp. nov.

#### ﻿ Romaleosyrphus nephelaeus

Taxon classificationAnimaliaDipteraSyrphidae

Moran & Thompson sp. nov.

2982381F-EC70-5B0D-9CD6-83F634465282

http://zoobank.org/E32DF62B-3528-4C3B-8C5F-5B58631C6740

**Figure 11.**
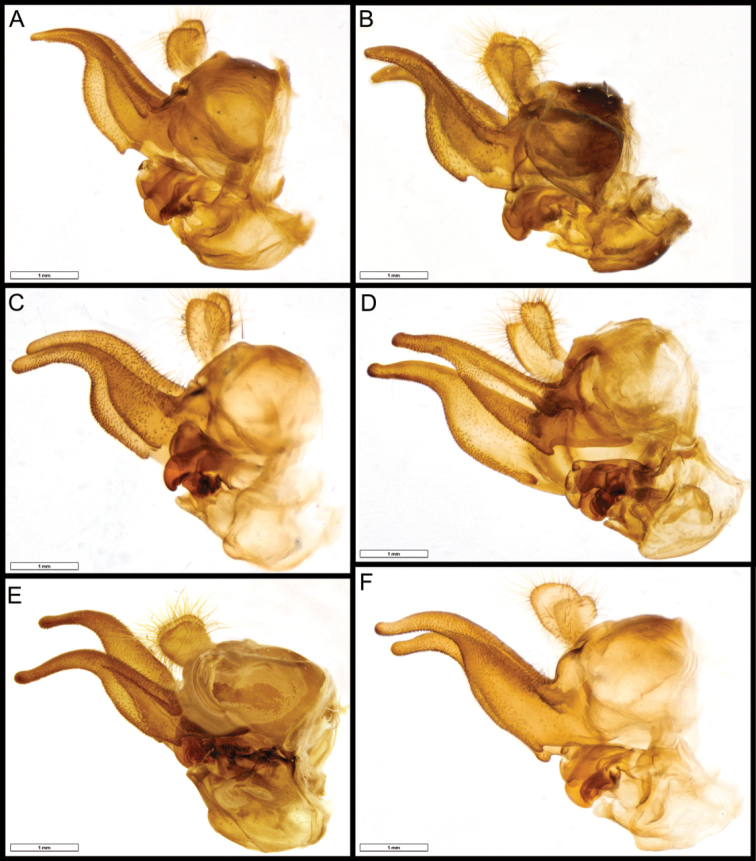
*Romaleosyrphus* male genitalia **A***Romaleosyrphusarctophiloides***B***Romaleosyrphussoletluna* sp. nov. **C***Romaleosyrphusnephelaeus* sp. nov. **D***Romaleosyrphusvockerothi* sp. nov. **E***Romaleosyrphusbigoti* sp. nov. **F***Romaleosyrphusvillosus*.

Figures 5C , 7C , 9C , 10C , 11C

##### Type locality.

**El Salvador**: Montecristo, 14.3664, -89.3842.

##### Types.

***Holotype*** male, pinned. Original label: “4 – 20 – 1978 // Monte Cristo // El Salvador, CA // D. R. Barger” “USNMENT // [BARCODE] // 01087036” (USNM). ***Paratypes***: **El Salvador**: Montecristo, 14.36639, -89.38417, D.R. Barger, 20.iv.1978, USNM_ENT1087030; ...USNM_ENT1087058; …USNM_ENT1087078 (1♂, USNM, 1♂ CNC, 1♂ RMNH); roadside, J.H. Davis, 22.iv.1977, USNM_ENT1087092 (1♂, USNM).

**Differential diagnosis.** Scutellum completely yellow pilose. Tergite II black pilose, except yellow pilose in anterolateral corners. Tergite III black pilose, although lateral margins mixed black and yellow. Tergite IV yellow pilose. Male hind tibia as in Fig. 9C. Male genitalia as in Fig. 11C.

##### Description.

**MALE.** Body length: 13.1–17.2 mm. Wing length: 9.2–12.1 mm.

***Head*.** Face shape as in Fig. 10C; face gold pruinose; gena yellow pilose posteriorly; anterior tentorial pit variable pilose: yellow or black; frons broad, ca. as long as broad at antenna, 2/3 as broad at vertex as at antenna, black pilose and silver-gold pruinose; vertex triangular, longer than broad, black pilose and brown pruinose; postocular setae black; occipital setae yellow; antenna reddish orange.

***Thorax.*** Matte black; postpronotum mixed black and yellow pilose; scutum yellow pilose along margins and black pilose medially; scutellum completely yellow pilose; postalar callus yellow pilose; proepimeron yellow pilose; posterior anepisternum yellow pilose; katepisternum yellow pilose posteriorly with broadly separated patches; metasternum variable pilose: black, yellow, or mixed black and yellow; anepimeron with anterior portion yellow pilose; lower calypter with long black pile.

***Legs.*** Coxae black; femora black except extreme apex of femora; remainder of legs reddish; hind trochanter tuberculate as in Fig. 8B; fore and mid-coxa black pilose; hind coxa mixed black and yellow pilose; fore femur black pilose, except small mix of yellow pile basally; mid femur black pilose, but with stretch of yellow pile on posterior side; hind femur black pilose; tibiae and tarsi black pilose; hind tibia as in Fig. 9C.

***Wing*.** Microtrichia absent from following areas: broad anterior margin of cell cua.

***Abdomen*.** Tergites shiny to subshiny black; tergite I with scattered, yellow pile medially, except with short black pile in lateral corners; tergite II black pilose, except yellow pilose in anterolateral corners; tergite III black pilose, except lateral margins mixed black and yellow; tergite IV yellow pilose; tergites not pruinose; sternites I-III yellow pilose and not pruinose; sternite IV black; pile of postabdomen mixed black and yellow pilose.

***Male genitalia*.** (Fig. 11C) Cercus yellowish brown, broader at apex, covered with long yellow pile; surstylus brown, ca. as long as hypandrium, broadened basally with apical half tapering and directed ventrally with a rounded apex, ventral margin concave, undulated; pile on dorsal surface of surstylus, increasing in length posteriorly; minute spines on ventral surface and apical 3/4 of lateral inner and outer surface.

**FEMALE.** Unknown.

##### Distribution.

El Salvador.

##### Habitat.

Central American montane forests ecoregion.

##### Etymology.

Named *nephelaeus*, after the Greek *nephele* (cloud), after the high elevation cloud forests in which this genus is found. It is a noun in apposition.

#### ﻿ Romaleosyrphus soletluna

Taxon classificationAnimaliaDipteraSyrphidae

Moran & Thompson sp. nov.

DBB7D01B-878A-5D7A-8224-1D567BB8621A

http://zoobank.org/F2961868-C818-47D9-9F7F-07A6916C1674

Figures 4C, D , 6C, E , 9D , 10D , 11B

Criorhina sp. Ståhls (2006): 25.
Romaleosyrphus sp. MZH Y247 Moran et al. (2021): 30.

##### Type locality.

**COSTA RICA**, Villa Mills, 9.564227, -83.707515, 3000m.

##### Types.

***Holotype*** male, pinned. Original label: “COSTA RICA S José // Villa Mills 3000m // 24.II.87 D. M. Wood” “USNMENT // [BARCODE] // 01261985” (CNC). ***Paratypes*: COSTA RICA**: **Cartago**: 11 mi. S.W. of Cartago, 9.730195, -84.034415, 1920m, C.D. Michner et al., 3.vii.1963, KMM0918 (1♀, USNM); **Guanacaste**: Est. Cacao. Guanacaste, 10.958528, -85.495649, 1200 to 1400m, Steve Marshall, 20.ii.1996, INBIOCRI002239730 (1♂, CNC); **Heredia**: Área de conservación Cordillera Volcánica Central, 9.555000, -83.670000, 1.ii.1990, R. Gerardo, INBIOCRI000154398 (1♂, INBIO); 15.iv.2002, Z. M. Ángel, INB0003945461; INB0003945468 (2♂, INBIO);10.132, -84.125, 21.iv.2003, Z. M. Ángel, INB0003702365 (1♂, INBIO); Cerro Chompipe, Res. Biol. Chompipe, 10.088, -84.071, 1900m, G. & M. Wood, 17.i.1999, CNC_DIPTERA249643 (1♂, CNC); ...2100m, J.F. Corrales, 1994, INBIOCRI001146848; ...INBIOCRI001146849 (2♂, USNM); Parque Nacional Braulio Carrillo, Estación Barva, 10.133492, -84.121242, 2500m, J.F. Corrales, ii.1990, INBIOCRI000167748 (1♂, EMEC); ...A. Fernández, iii.1990, INBIOCRI00019854 (1♂, USNM); ...G. Rivera & A. Fernández, iii.1990, INBIOCRI000169854 (1♂, USNM); ...x.1989, INBIOCRI000108632 (1♀, USNM); …xi.1989, INBIOCRI000139986 (1♂, CDFA); ...G. Rivera, ix.1989, INBIOCRI000111238 (1♀, USNM); **Puntarenas**: Área de conservación Arenal, 10.298, -84.793, 1.i.1993, O. Norman, INBIOCRI001369122 (1♂, INBIO); Est. La Casona, Res. Biol. Monteverde, 10.302815, -84.796543, 1520m, N. Obando, iii.1991, INBIOCRI001309535 (1♂, RMNH); Monteverde, Cerro Chomogo, 10.32689, -84.8058, 1800m, D.M. Wood, 22-30.viii.1996, CNC_DIPTERA249644 (1♂, CNC); Monteverde, 10.302815, -84.796543, 1500m, D.M. Wood, 24-28.ii.1991, USNM_ENT01261986 (1♂, USNM); Golfo Dulce, 3km SW. Rincón, 8.670722, -83.514359, 10m, H. Wolda, iii.1991, USNM_ENT1087008 (1♀, USNM); **San José**: Área de conservación La Amistad Pacífico, 9.555000, -83.670000, 13.i.1996, G. R. Billen, INBIOCRI002392420 (1♀, INBIO); 9.459000, -83.553000, 2.iii.1993, Z. M. Angel, INBIOCRI001305894 (1♀, INBIO); Cerro Muerte, 20 km S. Empalme, 9.566582, -83.749957, 2800m, Hanson, 11.vi.1990, USNM_ENT1087023 (1♀, USNM); **PANAMA**: **Chiriquí**: Guadalupe arriba, 8.871076, -82.550536, H. Wolda, 1.viii-4.ix.1984, USNM_ENT1087055 (1♂, USNM).

**Differential diagnosis.** Scutum entirely black pilose with at most only with a few anterolateral yellow pili on scutellum or mesonotum entirely yellow to rufous pilose. Male hind tibia as in Fig. 9D. Male genitalia as in Fig. 11B.

##### Description

**black morph.**

**MALE.** Body length: 13.8–15.3 mm. Wing length: 9.6–10.5 mm.

***Head*.** Face shape as in Fig. 10D; face gold pruinose; gena black pilose posteriorly; anterior tentorial pit black pilose; frons broad, ca. as long as broad at antenna, 2/3 as broad at vertex as at antenna, black pilose and gold pruinose; vertex triangular, longer than broad, black pilose and brown pruinose; postocular setae black; occipital setae black; antenna reddish orange.

***Thorax.*** Matte black; postpronotum mixed black and yellow pilose; scutum black pilose, except sometimes scattered yellow pile along lateral margins; scutellum black pilose, except with scattered yellow pile along posterior margin; postalar callus black pilose or mixed black and yellow pilose; proepimeron yellow pilose; posterior anepisternum yellow pilose; katepisternum yellow pilose posteriorly with broadly separated patches; metasternum mixed black and yellow pilose; anepimeron with anterior portion yellow pilose; lower calypter with long black pile.

***Legs.*** Coxae black; femora black except extreme apex of femora; remainder of legs reddish; hind trochanter tuberculate as in Fig. 8B; fore and mid-coxae black pilose; hind coxa mixed black and yellow pilose; fore femur black pilose, except small mix of yellow pile basally; mid femur black pilose, but with stretch of yellow pile on posterior side; hind femur black pilose; tibiae and tarsi black pilose; hind tibia as in Fig. 9D.

***Wing*.** Microtrichia absent in following areas: broad anterior margin of cell cua, cell br except along spurious vein and the part right below the start of cell r_2+3_;

***Abdomen*.** Tergites shiny to subshiny black; tergite I with scattered, yellow pile; tergite II with dense black pile medially and yellow pile on lateral sides; tergite III with black pile except mixed yellow pile anteromedially and yellow pile in anterolateral corners; tergite IV with black pile; tergites not distinctly pruinose; sternites I–III yellow pilose and not pruinose; sternite IV black pilose; pile of postabdomen black.

***Male genitalia*.** (Fig. 11B) Cercus yellowish brown, broader at apex, covered with long yellow pile. Surstylus brown, ca. 2 × as long as broad, broadened basally with apical half tapering, directed downward and with an acute apex, ventral margin concave, undulated; pile on dorsal surface of surstylus, increasing in length posteriorly; minute spines on ventral surface and apical 3/4 of lateral inner and outer surfaces.

##### Description

**rufous morph.**

**MALE.** Same as black morph except as follows.

***Head.*** Gena yellow pilose posteriorly; anterior tentorial pit yellow pilose; vertex rufous pilose; postocular setae rufous; occipital setae rufous.

***Thorax.*** Postpronotum rufous pilose; scutum rufous pilose; scutellum rufous pilose; postalar callus rufous pilose.

***Legs.*** Coxae yellow pilose; fore and mid femora yellow pilose; hind femur rufous pilose; tibiae and tarsi black pilose; metasternum yellow pilose.

***Abdomen*.** Tergite II with dense rufous pile medially and yellow pile on lateral sides; tergite III with rufous pile except mixed yellow pile anteromedially and yellow pile in anterolateral corners; tergite IV with rufous pile; sternites I–IV rufous pilose; pile of postabdomen rufous.

**FEMALE.** As male, except for usual sexual dimorphism; microtrichia on wing absent in following areas: middle third of cell r_1_, cell r_2+3_ along margin of vein R_2+3_ on the anterior third of cell, cell br except along spurious vein and the part right below the start of cell r_2+3_, all of cell cua except extreme posterior, ventral 2/3 of cell bm, cell cup along the margin of vein CuP in the posterior half, cell m_4_ from cross-vein m-cu to end of vein M_2_ and cell dm except for a thin line of microtrichia extending from cross-vein bm-m into middle of cell and the margins of cross-vein dm-m.

##### Distribution.

Costa Rica and Panama.

##### Habitat.

Talamancan montane forests (one specimen was collected in lowland rainforest).

##### Remarks.

Color morphs are considered to be intraspecific variation. No morphological differences were found outside of pile coloration in male genitalia or external characters. Additionally, these morphs are not associated with distinct COI haplotypes. It is difficult to argue in favor of interspecific variation without the addition of contradictory genetic evidence or fieldwork showing these morphs do not interbreed.

##### Etymology.

Named *soletluna*, a combination of the Latin words *sol*, for sun, and *luna*, for the moon. It is a reference to the duality of the color morphs in this species. It is a noun in apposition.

#### ﻿ Romaleosyrphus villosus

Taxon classificationAnimaliaDipteraSyrphidae

Bigot, 1882

D1E06A71-4032-5304-A57C-B040D107B6BC

Figs 4E , 6E , 8B , 9E , 10E , 11F

Romaleosyrphus villosus Bigot, 1882a: 159. –Bigot (1882b): cxxix. –Bigot (1883): 356. –Williston (1886): 300. **Type locality.** Mexico. [BMNH]
Crioprora villosa Williston, 1891: 73. –Aldrich (1905): 401. –Coquillett (1910): 528. –Kertész (1910): 291.
Criorhina villosa Thompson, 1976: 119.

##### Material examined.

**El Salvador**. Montecristo, 14.36639, -89.38417, 20.iv.1978, D.R. Barger, USNM_ENT1087039 (1♂, USNM); near Metapán, Montecristo, 14.383639, -89.385111, 2300m, 8-10. v.1971, S. Peck, CNC_Diptera142469 (1♂, CNC); **Honduras**. **Santa Bárbara**: Santa Bárbara 11.5 km S. & 5.6 km W. Peñas Blancas, 14.968983, -88.091211, 1870m, 20.vi.1994, R. Anderson, CNC_Diptera101960 (1♀, CNC); **Francisco Morazán**: San Juancito, 14.220280, -87.0675, 30.iii.1982, R. W. Jones, TAMU-ENTOX0290054 (1♀, TAMU); **Olancho**: Catacamas, 15.83333, -85.85139, 02.iii.1996, R. Cave, MZLU2014394 (1♀ MZLU); **Mexico**. **Chiapas**: Tzomtehuitz, near San Cristóbal, 16.833333, -92.633333, 19.v.1969, W.R.M. Mason, CNC_Diptera142472 (1♀, CNC).

##### Differential diagnosis.

Scutellum yellow pilose. Tergite II yellow pilose anteriorly and rufous pilose posteriorly. Tergite III rufous pilose anteriorly and black pilose posteriorly. Tergite IV dominantly black pilose. Male hind tibia as in Fig. 9E. Male genitalia as in Fig. 11F.

##### Redescription.

**MALE.** Body length: 13.8–15.3 mm. Wing length: 9.9–10.5 mm.

***Head*.** Face shape as in Fig. 10E; face silver or gold pruinose; gena black pilose posteriorly; anterior tentorial pit black pilose; frons broad, ca. as long as broad at antenna, 2/3 as broad at vertex as at antenna, black pilose and silver-gold pruinose; vertex triangular, longer than broad, black pilose and brown pruinose; postocular setae black; occipital setae black; antenna reddish orange.

***Thorax.*** Matte black; postpronotum variable pilose: black or mixed black and yellow; scutum black pilose; scutellum yellow pilose; postalar callus yellow pilose; proepimeron yellow pilose; posterior anepisternum yellow pilose; katepisternum yellow pilose posteriorly with broadly separated patches; metasternum variable pilose: black, yellow, or mixed black and yellow; anepimeron with anterior portion yellow pilose; lower calypter with long black pile.

***Legs.*** Coxae black; femora black except extreme apex of femora; remainder of legs reddish; hind trochanter tuberculate as in Fig. 8B; fore and mid-coxae black pilose; hind coxa mixed black and yellow pilose; fore femur black pilose, except small mix of yellow pile basally; mid femur black pilose, but with stretch of yellow pile on posterior side; hind femur black pilose; tibiae and tarsi black pilose; hind tibia as in Fig. 9E.

***Wing*.** Microtrichia absent from following areas: broad anterior margin of cell cua;

***Abdomen*.** Tergites shiny to subshiny black; tergite I with scattered, yellow pile; tergite II with dense yellow pile on anterior half and rufous pile on posterior half; tergite III with dense rufous pile on anterior third and black pile on posterior 2/3; tergite IV with black pile; grey pruinosity as follows: tergite I pruinose posteriorly, all of tergite II, tergite III anteriorly; sternites I–III yellow pilose and not pruinose; sternite IV black pilose; pile of postabdomen black.

***Male genitalia*.** (Fig. 11F) Cercus yellowish brown, broader at apex, covered with long yellow pile; surstylus brown, ca. 3 × as long as broad, broadened basally with apical third tapering, directed ventrally and with a rounded apex, ventral margin concave, undulated; pile on dorsal surface of surstylus, increasing in length posteriorly; minute spines on ventral surface and apical 3/4 of lateral inner and outer surfaces.

**FEMALE.** As male, except for usual sexual dimorphism. Microtrichia on wing absent in following areas: broad anterior margin of cell cua, medial area of cell bm, anteriorly in cell dm.

**Distribution.** El Salvador, Honduras, and Mexico.

##### Habitat.

Central American pine-oak forests ecoregion.

#### ﻿ Romaleosyrphus vockerothi

Taxon classificationAnimaliaDipteraSyrphidae

Moran & Thompson sp. nov.

0DD97F0B-CDCD-5DDD-AD4E-FD9EA137AE6B

http://zoobank.org/E2735672-CD68-4C91-9E5A-71BE2ED6CF8F

Figs 4B , 6B , 10F , 11D

##### Type locality.

**Mexico**: **Durango**: 14 miles Southwest of El Salto, 23.702771, -105.564051, 2438m.

##### Types.

***Holotype*** male, pinned. Original label: “MEX. Dgo. 14 mi. SW. // El Salto, 8000’ // 26 June 1964 // W. R. M. Mason” “CNCDIPTERA // # 142468” (CNC).

***Paratypes*: Mexico**: **Durango**: 14 miles Southwest of El Salto, 23.702771, -105.564051, 2438m, J.F. McAlpine, 26.vi.1964, CNC_Diptera142470 (1♂, RMNH); 30.vi.1964, CNC_Diptera142471 (1♂, CNC); 24 mi. W. La Ciudad, 23.723225, -106.065172, 2133m, J.F. McAlpine, 2.vii.1964, USNM_ENT01261987 (1♂, USNM).

##### Differential diagnosis.

Scutellum completely yellow pilose. Tergites II and III extensively rufous to yellow pilose. Tergite IV dominantly black pilose. Hind trochanter tuberculate in male. Male hind tibia as in Fig. 9E. Male genitalia as in Fig. 11D.

##### Description.

**MALE.** Body length: 13.8–14.5 mm. Wing length: 9.8–10.5 mm.

***Head*.** Face shape as in Fig. 10F; face silver or gold pruinose; gena black pilose posteriorly; anterior tentorial pit black pilose; frons broad, ca. as long as broad at antenna, 2/3 as broad at vertex as at antenna, black pilose and silver-gold pruinose; vertex triangular, longer than broad, black pilose and brown pruinose; postocular setae black; occipital setae variable: yellow or black; antenna reddish orange.

***Thorax.*** Matte black; postpronotum variable pilose: black or mixed black and yellow; scutum either yellow pilose along margins with black pile medially, or completely black pilose; scutellum completely yellow pilose; postalar callus variable pilose: yellow, black, or mixed black and yellow; proepimeron black pilose; posterior anepisternum yellow pilose; katepisternum yellow pilose posteriorly with broadly separated patches; metasternum variable pilose: black, yellow or mixed black and yellow; anepimeron with anterior portion yellow pilose; lower calypter with long black pile.

***Legs.*** Coxae black; femora black except extreme apex of femora; remainder of legs reddish; hind trochanter tuberculate as in Fig. 8B; fore and mid-coxae black pilose; hind coxa mixed black and yellow pilose; fore femur black pilose, except small mix of yellow pile basally; mid femur black pilose, but with stretch of yellow pile on posterior side; hind femur black pilose; tibiae and tarsi black pilose; hind tibia as in Fig. 9E.

***Wing*.** Wing completely microtrichose.

***Abdomen*.** Tergites shiny to subshiny black; tergite I with scattered, yellow pile medially, except with short black pile in lateral corners; tergite II with dense yellow pile; tergite III with dense pile which is yellow anteromedially and rufous on the remainder; tergite IV with dense black pile, although sometimes red pilose medially; tergites I–III pruinose; sternites I–III yellow pilose and not pruinose; sternite IV variable: black or rufous pilose or some mix of the two; pile of postabdomen black or rufous.

***Male genitalia*.** (Fig. 11D) Cercus yellowish brown, broader at apex, covered with long yellow pile; surstylus brown, distinctly longer than hypandrium, broadened basally with apical third tapering and not distinctly curved with a rounded apex, ventral margin concave, undulated; pile on dorsal surface of surstylus, increasing in length posteriorly; minute spines on ventral surface and apical 3/4^th^ of lateral inner and outer surface.

**FEMALE.** Unknown.

##### Distribution.

Mexico.

##### Habitat.

Sierra Madre Occidental pine-oak forests.

##### Etymology.

Named after J. R. Vockeroth in honor of his lifetime of work on Syrphidae and who was the first to recognize characters distinguishing this species from the sympatric *Romaleosyrphusarctophiloides* many years ago.

#### ﻿ Romaleosyrphus woodi

Taxon classificationAnimaliaDipteraSyrphidae

Moran sp. nov.

67C94D52-266F-5199-B1FA-2C7EE5D4323E

http://zoobank.org/4DD32215-AD71-459C-9F57-CAFA17A3EAD1

Figs 5A , 7A

##### Type locality.

**Mexico**: **Chiapas**: 16 mi. west of San Cristóbal, Chiapas, 16.7262, -92.8802.

##### Types.

***Holotype*** female, pinned. Original label: “San Christobal. // 16 mi W., Chiapas // MEX., VII-16-57” “UC Berkeley // EMEC // 354663 // [BARCODE]” (EMEC).

**Differential diagnosis.** Scutellum only partly yellow pilose, black pilose anteromedially. Tergite II black pilose in posterolateral corners. Tergite III black pilose except yellow pilose anteromedially. Cell r_2+3_ bare along margin of vein R_4+5_ starting from 2/5 of length of cell and ending at cross-vein r-m.

**Description. FEMALE.** Body length: 13.1 mm. Wing length: 9.1 mm.

***Head*.** Face non-pruinose; anterior tentorial pit black pilose; frons, black pilose and brown pruinose on lateral margins; vertex black pilose and brown pruinose; postocular setae black; occipital setae black; antenna reddish orange.

***Thorax.*** Matte black; postpronotum mixed black and yellow pilose; scutum black pilose, except yellow pilose along lateral margins; scutellum yellow pilose, except black pilose anteromedially; postalar callus yellow pilose; proepimeron black pilose; posterior anepisternum yellow pilose; katepisternum yellow pilose posteriorly with broadly separated patches; metasternum mixed black and yellow pilose; anepimeron with anterior portion yellow pilose; lower calypter with long black pile.

***Legs.*** Coxae black; femora black except extreme apex of femora; remainder of legs reddish; fore and mid-coxae black pilose; hind coxa mixed black and yellow pilose; fore femur black pilose, except small mix of yellow pile basally; mid femur black pilose, but with stretch of yellow pile on posterior side; hind femur black pilose; tibiae and tarsi black pilose.

***Wing*.** Microtrichia absent in following areas: cell c along margin of vein Sc running from 2/5 of length and ending at 4/5 of length of the cell, anterior 1/5 of cell r_1_, r_2+3_ along margin of vein R_4+5_ starting from 2/5 of length and ending at cross-vein r-m, cell br except along spurious vein and the part right below the start of cell r_2+3_, all of cell cua except extreme posterior, cell bm, cell cup along the margin of vein CuP in the anterior third of cell, cell m_4_ from cross-vein m-cu to end of vein M_4_ and cell dm in ventral 1/3 of cell and along broad margin following vein M_2_.

***Abdomen*.** Tergites shiny to subshiny black; tergite I with scattered, yellow pile medially, except with short black pile in lateral corners; tergite II with dense yellow pile which runs diagonally from anterolateral corner until it reaches the posterior margin at a point which is ca. at 1/3 of the width of the tergite, remainder of tergite is black pilose; tergite III with black pile except mixed yellow pile anteromedially; tergite IV with black pile; tergites not distinctly pruinose; sternites I–III yellow pilose and not pruinose; sternite IV black pilose; pile of postabdomen black.

**MALE**. Unknown.

##### Distribution.

Mexico.

##### Habitat.

Central American pine-oak forests ecoregion.

**Remarks.** The specimen failed to barcode. Most similar in appearance to *Romaleosyrphusdrysus* sp. nov. but *R.woodi* sp. nov. differs in having a scutellum which is only partly yellow pilose, instead having black pile anteromedially. Additionally, cell r_2+3_ is bare along the margin of vein R_4+5_ starting from 2/5 the length of cell and ending at cross-vein r-m.

##### Etymology.

Named after dipterologist Monty Wood to honor his passion for flies and whose collecting trips throughout Central and South America provided many critical Syrphidae for this as well as other future studies.

### ﻿Species concepts and DNA barcoding

DNA barcode data (5’ end of the COI) were collected for eight of nine morphospecies to provide a database to assist with future identifications of all life stages. Complete barcodes were obtained for all species except *R.woodi* sp. nov. Additional sequences for *Romaleosyrphus* were obtained from the BOLD database.

The rufous and black morphs of *R.soletluna* sp. nov. are not differentiated by COI haplotype showing that coloration should be considered intraspecific variation. The barcode differs by an average pairwise (p) distance of 3.04% from its nearest neighbor *Romaleosyrphusbigoti* sp. nov. It has a maximum intraspecific variation of 0.93% and an average of 0.56%.

*Romaleosyrphusarctophiloides* is related to the *R.villosus* complex of species (*R.villosus*, *R.vockerothi* sp. nov., *R.bigoti* sp. nov.) with the barcode 2.34% different from the nearest neighbor *Romaleosyrphusvillosus*. This is the only known species of *Romaleosyrphus* in which males lack a tubercle on the hind trochanter.

Separation of *R.bigoti* sp. nov. and *R.vockerothi* sp. nov. species from *R.villosus* is supported by DNA barcoding. The barcoded types are 1.52% and 1.55% different from their closest neighbor, respectively. This distance is nearly twice as high as the maximum intraspecific variation seen in *R.soletluna* sp. nov. (0.93%) and *Romaleosyrphusnephelaeus* sp. nov.(0.97%). Morphological differences are found in the shape of the male genitalia as well as the shape of the male hind tibia.

The nearest neighbor of *R.argosi* sp. nov. is *Romaleosyrphusdrysus* sp. nov. with the COI barcodes diverging by 3.05%. The nearest neighbor of *Romaleosyrphusdrysus* sp. nov. is *Romaleosyrphusnephelaeus* sp. nov. with the COI barcodes diverging by an average of 2.85%. These distinct barcodes along with the unique pile coloration patterns of *Romaleosyrphusargosi* sp. nov. and *Romaleosyrphusdrysus* sp. nov. support the recognition of these specimens as new species.

While the type of *R.woodi* sp. nov. failed to produce a barcode, morphological evidence was found in favor of its recognition as a distinct species. The species is most similar in appearance to *Romaleosyrphusdrysus* sp. nov. but differs in having a scutellum which is only partly yellow pilose, instead having black pile anteromedially. Additionally, cell r_2+3_ is bare along the margin of R_4+5_ starting from 2/5 the length of cell and ending at cross-vein r-m.

## ﻿Discussion

Moran et al. (2021) resurrected *Romaleosyrphus* placing ‘*Romaleosyrphus* sp. MZH Y247’, now known as *Romaleosyrphussoletluna* sp. nov., sister to the genus *Matsumyia*.

In concordance with the neighbor-joining analysis, as well as the multi-gene analysis of Moran et. al (2021), morphological evidence supports the monophyletic origin of these Neotropical species, their relationship with *Matsumyia* and also their separation. The two genera share several characters and are distinguished from members of *Criorhina* and *Sphecomyia* by: holoptic males, a proximal ventral half of vein C with setae, a broad intersection of vein R_1_ with vein C, and appressed hair on the abdomen. Additionally, *Romaleosyrphus* is further distinguished from *Matsumyia* by a distal R_4+5_ longer than cross-vein h. All species of *Matsumyia* examined, as part of an upcoming revision of the genus, however, had a distal R_4+5_ shorter than cross-vein h. *Romaleosyrphus* stat. rev. is therefore redefined to represent the monophyletic unit of species within Criorhinina which possess these five character states.

**Figure 12.**
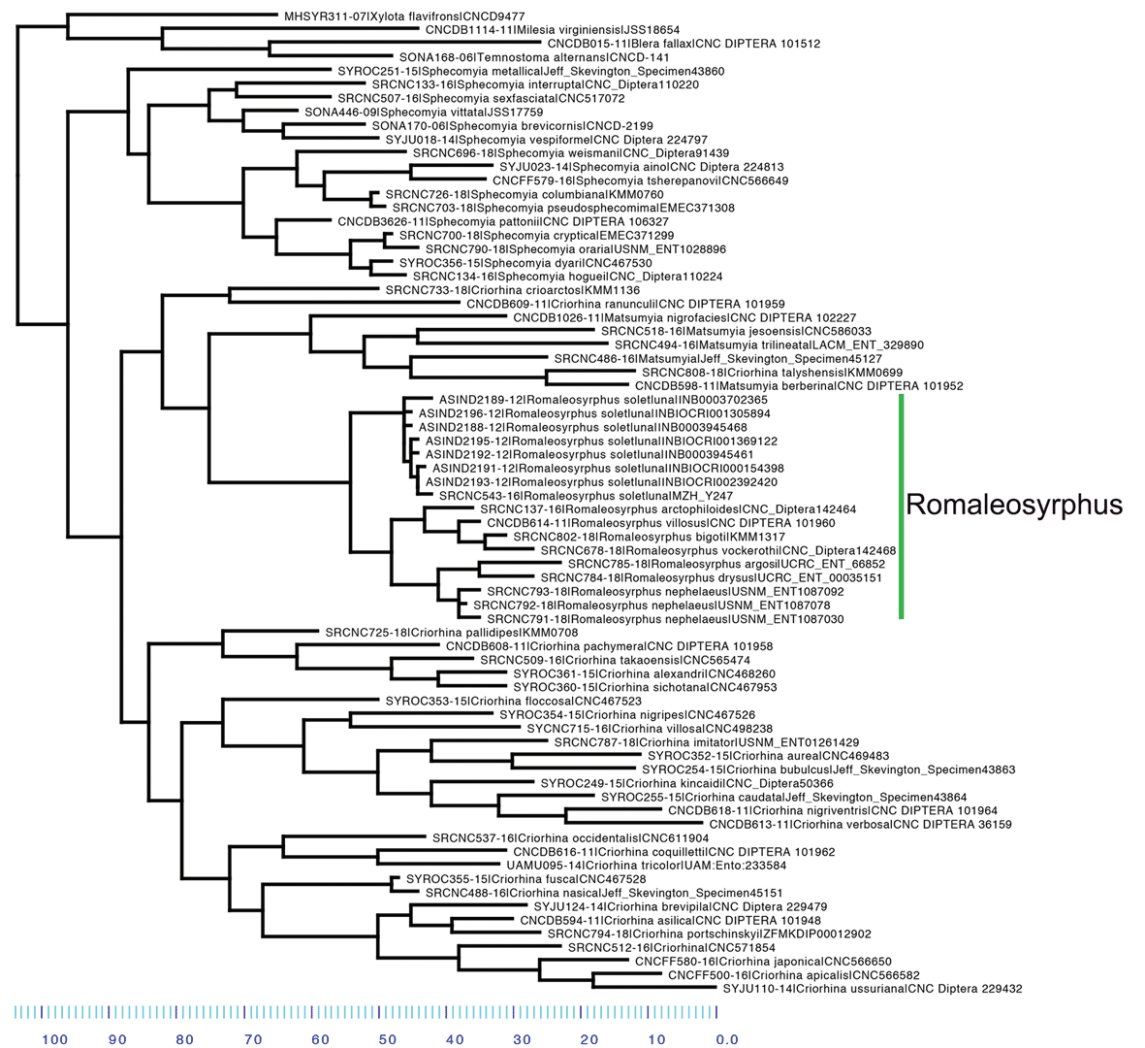
Neighbor-Joining tree based on the barcode region of the mitochondrial cytochrome *c* oxidase subunit I gene.

Hampered by the rarity of *Romaleosyrphus* and the age of most specimens, more than one sequence was obtained for only two species and neither showed a high degree of intraspecific variation (Fig. 12). DNA barcodes reveal *R.soletluna* sp. nov. is dimorphic in pile coloration and these morphs are not associated with distinct COI haplotypes. The genetic distance between *Romaleosyrphus* species is lower than between species of most other Criorhinina genera. For example, *Matsumyia* species show a much higher degree of species differentiation both for DNA barcodes and external morphological characters. It is possible that *Romaleosyrphus* diversified more recently. This may explain their less divergent intrageneric morphology and it would be worth investigating whether speciation coincided with the arrival of *Bombus* in Central America. Fresh material and more markers are needed to test these questions.

The discovery of the larvae of *Romaleosyrphus* would add critical biological knowledge about this genus and their microhabitats. Most likely, immatures live on decaying roots akin to the larvae of *Matsumyiaberberina* (Fabricius, 1805), the most closely related species for which larvae is known, as also do larvae of some *Criorhina* species (Speight, 2020). Alternatively, larvae may be associated with rot-holes, sap-runs, or decaying wood in general as in other *Criorhina* species (Speight, 2020).

Moving forward, the authors suspect additional *Romaleosyrphus* species have yet to be discovered considering their apparent rarity and that their high elevation cloud forest habitat is highly conducive to speciation (Bruijnzeel, 2010). Currently, the center of diversity of the genus appears to be either the Central American montane forest ecoregion or the Central American pine-oak forest ecoregion, with three species each. One species each is known from the Sierra Madre Occidental pine-oak forest, the Talamancan montane forests and the Trans-Mexican Volcanic Belt pine-oak forests.

No species have been recorded from several similar ecoregions: Oaxacan, Chiapas, Chimalapas, and the Veracruz montane forests, along with the Sierra Madre de Oaxaca, Sierra Madre Oriental, Sierra Madre del Sur, and the Sierra de la Laguna pine-oak forests. It is also uncertain if the genus extends into the montane pine-oak forest ecoregions of South America. Additional collecting efforts focused on these ecoregions are necessary to discover the extent of *Romaleosyrphus* biodiversity.

## ﻿Conclusion

Based upon molecular and morphological evidence we redefine *Romaleosyrphus* stat. rev. as the monophyletic unit of species within Criorhinina which possesses holoptic males, a proximal ventral half of vein C with setae, a broad intersection of vein R_1_ with vein C, a distal R_4+5_ longer than cross-vein h, and appressed pile on the abdomen. This requires the transfer of *Romaleosyrphusvillosus* (Bigot, 1882a) comb. nov. and *Romaleosyrphusarctophiloides* (Giglio-Tos, 1892) comb. nov. to *Romaleosyrphus*.

## Supplementary Material

XML Treatment for Romaleosyrphus

XML Treatment for Romaleosyrphus arctophiloides

XML Treatment for Romaleosyrphus argosi

XML Treatment for Romaleosyrphus bigoti

XML Treatment for Romaleosyrphus drysus

XML Treatment for Romaleosyrphus nephelaeus

XML Treatment for Romaleosyrphus soletluna

XML Treatment for Romaleosyrphus villosus

XML Treatment for Romaleosyrphus vockerothi

XML Treatment for Romaleosyrphus woodi

## Supplementary materials

Supplementary material 1 *Romaleosyrphus* material examined Data type species data) https://binary.pensoft.net/file/619724This dataset is made available under the Open Database License (http://opendatacommons.org/licenses/odbl/1.0/). The Open Database License (ODbL) is a license agreement intended to allow users to freely share, modify, and use this Dataset while maintaining this same freedom for others, provided that the original source and author(s) are credited.Kevin M. Moran, Jeffrey H. Skevington

## References

[B1] Aldrich JM (1905) A catalogue of North American Diptera (or two-winged flies) VL – XLVI. Smithsonian Miscellaneous Collections 46 (2): I–680. 10.5962/bhl.title.1681

[B2] Bigot JMF (1882a) Descriptions de genres et espèces inédits de Syrphidés (3^eme^ partie). Bulletin de la Société Entomologique de France 14: 159–163. https://biodiversitylibrary.org/page/4241989

[B3] Bigot JMF (1882b) Diagnoses de genres et espèces inédits de Syrphidés. 3^eme^ partie. Annales de la Société entomologique de France (6)2: 128–129. https://biodiversitylibrary.org/page/8998058

[B4] Bigot JMF (1883) Diptères nouveaux ou peu connus. 21e partie, XXXII: Syrphidi (1^ere^ partie). Annales de la Société entomologique de France (6) 3: 221–258. [1883.10.31] https://biodiversitylibrary.org/page/32548784

[B5] Bruijnzeel LA Scatena FN Hamilton L (2010) Tropical Montane Cloud Forests: Science for Conservation and Management. Cambridge University Press, 768 pp. 10.1017/CBO9780511778384

[B6] Coquillett DW (1910) The Type-Species of the North American Genera of Diptera. Proceedings of the United States National Museum 37: 499–622. 10.5479/si.00963801.37-1719.499

[B7] Cumming JM Wood DM (2017) Adult morphology and terminology. In: Kirk-Spriggs AHS Sinclair BJ (Eds) Manual of Afrotropical Diptera.Volume 1. Introductory chapters and keys to Diptera families. Suricata 4. South African National Biodiversity Institute, Pretoria, 21–65.

[B8] Folmer O Black M Hoeh W Lutz R Vrijenhoek R (1994) DNA primers for amplification of mitochondrial cytochrome *c* oxidase subunit I from diverse metazoan invertebrates. Molecular Marine Biology and Biotechnology 3: 294–299. 7881515

[B9] Gibson JF Kelso S Jackson MD Kits JH Miranda GFG Skevington JH (2011) Diptera-specific polymerase chain reaction amplification primers of use in molecular phylogenetic research. Annals of the Entomological Society of America 104: 976–997. 10.1603/AN10153

[B10] Giglio-Tos E (1892) Diagnosi di nuove specie di Ditteri. VI. Bollettino dei musei di zoologia ed anatomia comparata della R. Università di Torino 7(123): 1–7. 10.5962/bhl.part.12593

[B11] Giglio-Tos E (1893) Part I. Stratiomyidae – Syrphidae. Torino, 72 pp. [71 pl.]

[B12] Hajibabaei M deWaard JR Ivanova NV Ratnasingham S Dooh RT Kirk SL Mackie PM Hebert PDN (2005) Critical factors for assembling a high volume of DNA barcodes. Philosophical Transactions of the Royal Society B 360: 1959–1967. 10.1098/rstb.2005.1727 PMC1609220 16214753

[B13] Katoh K Standley DM (2013) MAFFT multiple sequence alignment software version 7: improvements in performance and usability. Molecular Biology and Evolution 30: 772–780. 10.1093/molbev/mst010 23329690 PMC3603318

[B14] Kertész K (1910) Catalogus dipterorum hucusque descriptorum. VII. Museum Nationale Hungaricum, Budapest, 470 pp. 10.5962/bhl.title.5147

[B15] Moran KM Skevington JH Kelso S Mengual X Jordaens K Young AD Ståhls G Mutin V Bot S van Zuijen M Ichige K van Steenis J Hauser M van Steenis W (2021) A multi-gene phylogeny of the eristaline flower flies (Diptera: Syrphidae), with emphasis on the subtribe Criorhinina. Zoological Journal of the Linnean Society 2021: zlab006. 10.1093/zoolinnean/zlab006

[B16] Rye EC (1884) Index to genera and subgenera recorded as new in this volume. Zoological Record 19: 1–11.

[B17] Ståhls G (2006) Placement of *Cacoceria* and phylogenetic relationships of the xylotine genera of the tribe Milesiini (Diptera, Syrphidae: Eristalinae) based on molecular characters. Zootaxa 1171: 17–29. 10.11646/zootaxa.1171.1.2

[B18] Speight MCD (2020) Species accounts of European Syrphidae, 2020. Syrph the Net, the database of European Syrphidae (Diptera) 104: 1–314.

[B19] Swofford DL (2001) PAUP*. Phylogenetic analysis using parsimony (*and Other Methods). 4.0b10 edn. Sinauer Associates, Inc., Sunderland.

[B20] Thompson FC (1999) A key to the genera of the flower flies (Diptera: Syrphidae) of the Neotropical Region including descriptions of new genera and species and a glossary of taxonomic terms. Contributions on Entomology, International 3: 322–378.

[B21] Thompson FC Vockeroth JR Sedman YS (1976) Family Syrphidae. A catalogue of the Diptera of the Americas south of the United States. Volume 46. Museu de Zoología, Universidade de São Paulo, São Paulo, 195 pp. 10.5962/bhl.title.110114

[B22] Williston SW (1886) Synopsis of the North American Syrphidae. Bulletin of the United States National Museum Vol. 31, 335 pp. 10.5962/bhl.title.40963

[B23] Williston SW (1891) Fam. Syrphidae. In: Godman FD Salvin O (Eds) Biologia Centrali-Americana – Zoologia – Insecta – Diptera, Volume 3.Porter RH, London, 1–56. [2 pls] 10.5962/bhl.title.730

